# Polycystic Ovary Syndrome and Psychotic Disorder

**DOI:** 10.3389/fpsyt.2020.00543

**Published:** 2020-06-10

**Authors:** Larissa Doretto, Flora Chaves Mari, Ana Cristina Chaves

**Affiliations:** ^1^First Episode Psychosis Program, Department of Psychiatry, Universidade Federal de São Paulo (UNIFESP), Sao Paulo, Brazil; ^2^Santa Casa Medical School of Sao Paulo, Sao Paulo, Brazil

**Keywords:** polycystic ovary syndrome, psychotic disorder, women, antipsychotic drugs, schizophrenia

## Abstract

Polycystic ovary syndrome (PCOS), a disease that usually emerges during adolescence, is characterized by hormonal imbalance and ovarian dysfunction. The prevalence can vary between 5.6 to 21.3% in women and 6% in adolescent girls. This discrepancy is related to the population studied and the diagnostic criteria used. The underlying pathophysiology of PCOS is not fully understood, but it can lead to a number of co-morbidities, including hypertension, diabetes, dyslipidemia, and also, mental health disorders. Clinical and preclinical data indicate neuroendocrine involvement with dysfunction in gamma-Aminobutyric acid (GABA) signaling and neuronal androgen receptors that might reduce hypothalamic sensitivity and lead to an impairment of estradiol and progesterone feedback. Based on these assumptions, the aims of this paper are to review the association of PCOS and psychotic disorders in order to address the burden of women comorbid for both conditions.

## Introduction

Polycystic ovary syndrome (PCOS) is among the most common endocrine disorders, affecting 5.6–21.3% of women of reproductive age worldwide (1, 2). It is a heterogenous clinical condition, with a range of different phenotypes, a clinical reality that results in divergent opinions regarding diagnosis and treatment. International guidelines have been developed with the aim of integrating the best available evidence on diagnosis, assessment, and treatment and improving clinical care (2). These guidelines highlight the necessity to avoid over diagnosis, especially in adolescents. They emphasize the uncertainty of the natural history of this syndrome and its clinical implications. They point to hyperandrogenism, ovulatory dysfunction, and polycystic ovaries (as seen on ultrasound) as the key diagnostic features. Other potential features mentioned are: menstrual irregularity, subfertility, obesity, hirsutism, acne, and abnormal biochemistry, namely elevations of serum testosterone, androstenedione, luteinizing hormone, and insulin. The guidelines warn that affected women are at increased risk for hypertension, dyslipidemia, insulin resistance, glucose intolerance, type 2 diabetes, coagulation disorders, as well as cardiovascular morbidity and mortality.

Psychiatric symptoms such as anxiety and depression are additional common features of the syndrome (3, 4), but they may be unrecognized by the treating physician. On the other hand, physicians who see patients for psychological problems may not ask about features of PCOS, or if they see clinical signs such as obesity, acne, and hirsutism, may automatically attribute them to the effects of psychiatric medications. Publications on comorbidity between PCOS and severe psychiatric disorders are more frequently seen in the gynecology and endocrinology literature than in papers on psychiatry. Lack of recognition of the co-existence of PCOS and psychiatric syndromes impacts negatively on affected women by delaying appropriate treatment. A large population-based study from Sweden found that 22.4% of the 22,385 women participants with PCOS had received at least one lifetime psychiatric diagnosis (5). When compared to a matched comparison sample, these women showed a higher prevalence not only of depression and anxiety, but also of less common disorders, such as bipolar disorder, schizophrenia spectrum disorder, eating disorder, and personality disorder.

There are several competing theories regarding the etiology of PCOS, but there is a consensus that the syndrome results from multiple causes. Significant interactions are likely among genetic and epigenetic factors, primary ovarian abnormalities, and neuroendocrine alterations (6). Recent evidence shows that neuroendocrine brain circuits, particularly the hypothalamic GnRH neuronal, are involved in the etiology of at least some forms of PCOS (7).

Similar neuroendocrine dysfunctions of the hypothalamic-pituitary axis are found in psychotic disorder (5). Few studies, however, have assessed the impact on clinical, course and prognosis of comorbid PCOS and psychosis. The aim of this paper, therefore, is to review this literature starting with the variety of theories explaining the pathophysiology of PCOS, followed by the consequences of the simultaneous occurrence of PCOS and psychosis, the role of antipsychotic medications, recommendations, and conclusion.

## Methods

This review is based on papers retrieved from PubMed searches using the following terms: “psychosis”, “schizophrenia”, “affective psychosis”, “antipsychotics”, “polycystic ovary syndrome”, “menstrual dysfunction”, “hyperandrogenism”. First, abstracts in English from papers published from 2005 to 2019 were assessed to evaluate their relevance to the combined clinical effect of the two conditions, psychosis and PCOS. The final inclusion/exclusion of articles was not based on a protocol developed before as in a systematic review.

### Theories About the Pathophysiology of PCOS

Based on a recent extensive review (8), we summarize in this section recent theories about the pathophysiology of PCOS. PCOS begins during the pubertal years, but the diagnosis is usually made later in life once the disorder has become relatively more severe. Ovarian dysfunction is thought to be caused by an impairment in the feedback loop of the steroid-hormone gonadotropin-releasing hormone (GnRH) produced in the hypothalamus. While there are other biologic systems and interconnected signaling networks also involved in the pathophysiology of PCOS, these latter networks may not be impaired in all affected women (6, 8)

**Figure 1:**
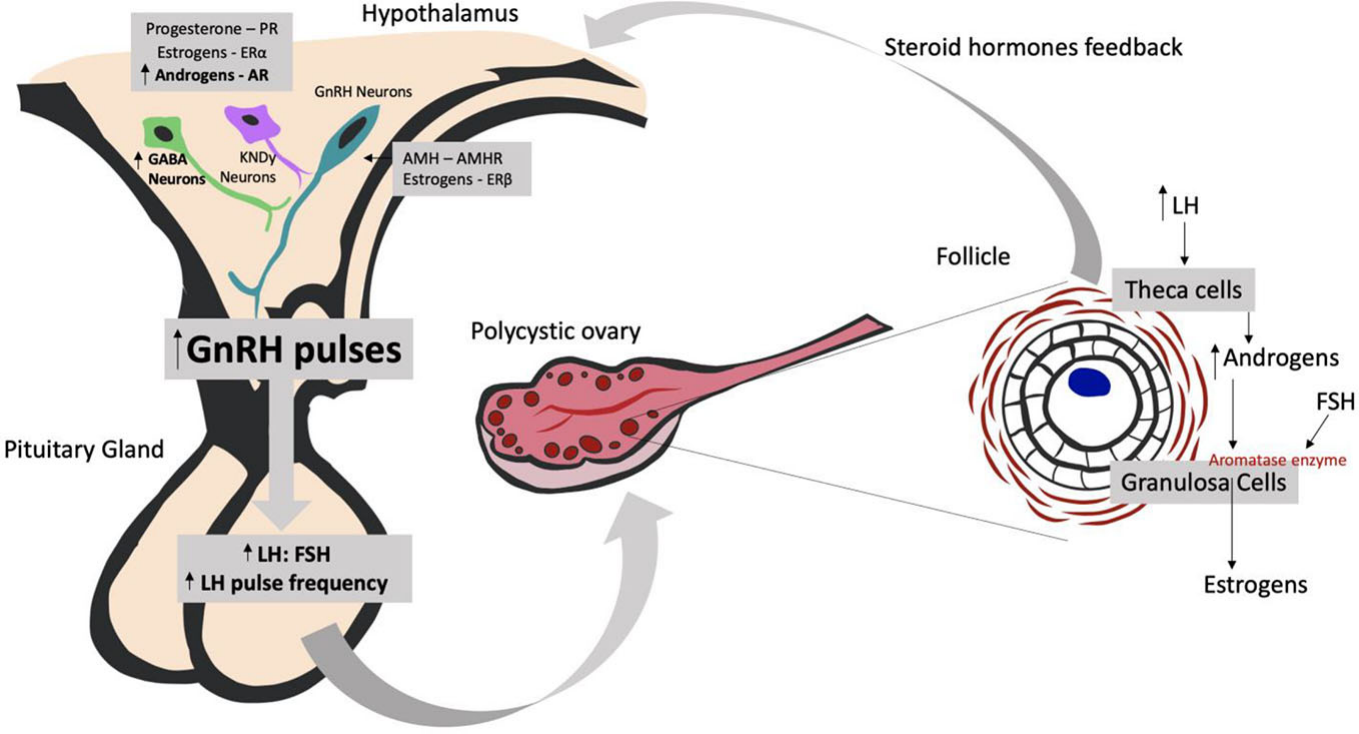
Understanding the neuroendocrine effects of the hyperandrogenic environment on PCOS development. AMH, Anti-Müllerian Hormone; AMHR, Anti-Müllerian Hormone Receptor; AR, Androgen Receptor; ERα, Estrogen Receptor α; ERβ, Estrogen Receptor β; FSH, Follicle-stimulating Hormone; GnRH, Gonadotropin-releasing Hormone; GABA neurons, gamma-Aminobutyric acid neurons; KNDy neurons, kisspeptin/dynorphin/neurokinin B neurons; LH, Luteinizing Hormone.

In healthy women, the frequency of GnRH pulses in the hypothalamus regulate the pulsatile release of luteinizing hormone (LH) and follicle stimulating hormone (FSH) from the anterior pituitary gland: faster frequencies favor LH secretion and slower frequencies favor FSH release. In turn, LH and FSH secretion regulates the production of follicles and gonadal steroids in the ovary. The level of sex steroid hormones produced by the ovaries—estrogens, progestogens, and androgens—provides critical feedback to the hypothalamus and pituitary gland, thus regulating the degree of GnRH, LH, and FSH secretion. In PCOS, this physiological feedback loop is compromised, resulting in hyperactivity of the hypothalamus-pituitary-gonadal axis and an abnormally high LH : FSH ratio. This then impairs follicle generation in the ovary and interferes with the production of steroid hormones (6). Ovarian follicles remain in their immature state, a pre-ovulatory stage characterized by the cystic appearance of the ovary on ultrasound. The increased secretion of LH, acting on ovarian theca cells, stimulates the production of androgens. While androgens would normally be transformed into estradiol *via* an FSH-induced aromatase synthesized in ovarian granulosa cells (see **Figure 1**), in women with PCOS, this transformation is impaired due to the altered LH/FSH ratio. This leads to a state of hyperandrogenism (9). It is unclear whether the problem starts with a dysfunction in GnRH neurons in the hypothalamus or whether this occurs secondarily due to pathology of upstream neuronal systems (6).

#### Hyperandrogenism and KNDy Neurons

Some pre-clinical models tested in animals are trying to understand how neuronal systems are causing hyperandrogenism in PCOS. Studies that promote hyperandrogenic environments during pre-natal life and/or post-natal life in female mice found alterations in gamma-Aminobutyric acid (GABA) neurons in the medial basal hypothalamus and kisspeptin/neurokinin B/dynorphin neurons (KNDy neurons) in the arcuate nucleus (ARN) (9). These hypothalamic neurons have receptors for progesterone (PR), estradiol receptor α (ERα), and androgens (AR) and are responsible for the control of GnRH neurons and their feedback. As GnRH neurons have only ERβ receptor and not ERα, which is the most important receptor in estrogen activity, the investigators concluded that the presence of these receptors in kisspeptin neurons and in GABAergic neurons could be essential for modulating the negative feedback to GnRH neurons (see **Figure 1**). However, the findings concerning KNDy neurons are still controversial: in some models, KNDy neuronal population is high, in others, low or even not different at all. However, inhibition of KNDy neurons has shown to ameliorate the signs and symptoms of women with PCOS, by decreasing LH pulse frequency and LH and testosterone serum levels (10).

#### Hyperandrogenism and GABA

Based on clinical findings showing elevated concentrations of GABA in the cerebrospinal fluid of women with PCOS, animal studies have also addressed the involvement of GABA (9). A hyperandrogenic environment in female mice in prenatal life can cause an increase in the frequency and degree of GABAergic postsynaptic firing onto GnRH neurons. Unlike its function in other brain circuits, GABA seems to exert an excitatory effect on GnRH neurons attributable to their high chloride content. This leads to greater secretion of LH by the pituitary gland, as occurs in PCOS. GnRH antagonists prevent this effect (9, 11).

#### Hyperandrogenism and Anti-Mullerian Hormone

Normally, a dynamic equilibrium exists between growing and dormant follicles in the ovaries, regulated by a balance between androgens, anti-Müllerian hormone (AMH), and FSH. In PCOS, this balance is disrupted, leading to follicular arrest. It is known that women with PCOS have higher than normal serum levels of AMH, and that these levels remain high even during pregnancy (11). Tata et al. (11) conducted a study in which they classified 63 women with PCOS and 66 control women into lean or obese groups according to their body mass index. They found that lean pregnant women with PCOS had higher AMH levels than lean pregnant controls. However, there was no difference in AMH levels between PCOS pregnant obese women and controls. These findings led to the creation of a mouse model prenatally treated with AMH, which induced brain masculinization of female offspring that then showed neuroendocrine and reproductive PCOS-like features. It was therefore concluded that high AMH levels during pregnancy may be the cause of a hyperandrogenic environment that ultimately leads to PCOS pathology (11).

#### Hyperandrogenism and Androgen Receptors (AR)

When animal experiments compared female knock out mice with no brain AR with mice with no AR in the granulosa cells of the ovary, the mice with no brain AR showed some of the reproductive and metabolic features of PCOS, whilst the group with no ovarian AR showed all the features. Despite this difference, the conclusion reached was that hyperandrogenic changes in the brain came first and were, therefore, primarily responsible for what followed (12).

#### Hyperandrogenism and Microbiota

It has been recently discovered that gut microbiota plays a role in host sex differences (8). The composition of commensal microbes of males and females appears to diverge at puberty, thus implicating sex hormone levels. Torres et al. (13) have suggested that hyperandrogenism may play a direct role in altering the gut microbiome of women with PCOS. (14), studying rats, demonstrated a shift in the distribution of microbiota that was associated with sex hormone levels. In addition, they showed that fecal transplants were able to decrease androgen blood levels and increase estrogen levels, thereby improving estrus cycles in rat models of PCOS. Gut dysbiosis, therefore, may turn out to be an important contributing factor in the genesis of PCOS.

#### Classification of PCOS

The many disparate findings of animal studies mirror the multiple hypotheses that have been formulated to address the potential etiology of PCOS. In order to minimize controversy, guidelines to improve diagnosis and treatment have been developed (2). The following classification system has been introduced: phenotype A (hyperandrogenism + ovulatory disfunction + polycystic ovarian morphology); phenotype B (hyperandrogenism + ovarian dysfunction); phenotype C (hyperandrogenism + polycystic ovulatory morphology); phenotype D (ovarian dysfunction + polycystic ovulatory morphology). The guidelines recognize that PCOS is also a metabolic disorder, but do not incorporate insulin resistance into the diagnostic criteria because tests for insulin resistance show poor accuracy (2). The prevalence of psychological features is also acknowledged, and recommendations are made for the investigation and treatment of psychiatric illness associated with PCOS.

### Women With PCOS and a Psychotic Disorder

Estrogen appears to exert an antipsychotic effect. When levels decline, the emergence of psychotic symptoms is facilitated (15). For instance, several literature reviews suggest that, in women with chronic psychotic illness, symptoms are aggravated during the premenstrual period (16), after delivery (17) and at menopause (15).

PCOS women, might, therefore, be vulnerable to psychosis because they are exposed to long durations of high levels of unopposed estrogen as a result of infrequent ovulation (18). When they do ovulate, they experience a precipitous reduction in estrogen, mimicking a postpartum state. This could explain the vulnerability of women with PCOS to psychotic symptoms (19).

Women with psychosis often show menstrual irregularity or amenorrhea, attributable to the hyperprolactinemia induced by the use of antipsychotic drugs (15). Hyperprolactinemia can also interfere with fertility (20), a problem for up to 72% of PCOS women (21). Antipsychotic medications impact negatively on personal appearance because of associated weight gain, hirsutism, acne, dental problems, halitosis, alopecia, rash, tremor, stiff gait, unsightly mouth movements, voice changes, or incontinence (22, 23). Similar symptoms are associated with PCOS, leading to a negative body image (24), low self-esteem, perceived stigma (25, 26), and a high prevalence of anxiety and depression (27).

The high degree of symptom overlap between the two conditions may be what prevents the recognition of primary PCOS in psychiatric patients. Additionally, for women suffering from both conditions, these symptoms are all aggravated.

### The Role of Antipsychotic Medication

Besides leading to PCOS-like symptoms, the chronic administration of antipsychotics has been shown to exert a negative impact on gut microbiota (28–32), increasing the dysbiosis caused by PCOS. Indeed, Davey et al. (30) demonstrated that the administration of olanzapine for 3 weeks in rats induced identifiable alterations in the microbiome. Antipsychotics have antibacterial properties. Olanzapine, for instance, is able to completely inhibit the growth of E. coli (32). For women with both conditions, hyperandrogenism could, in this way, be aggravated by the use of antipsychotics.

Furthermore, the weight gain induced by antipsychotics affects more than appearance. Elevated body mass index and intra-abdominal adiposity predict insulin resistance and type 2 diabetes (T2DM) in patients treated for long periods of time (33, 34). Particular drugs (especially clozapine and olanzapine) are more likely than others to cause weight gain (35, 36). Compared to untreated psychiatric controls, Galling and colleagues (37) reported an incidence of T2DM three times higher in youth treated with antipsychotics for over three months. Antipsychotic-induced diabetes has been confirmed in animal models; both olanzapine and clozapine have been shown to decrease the plasma level of insulin and to cause hyperglycemia and insulin resistance in rats (16).

Women with PCOS and without psychosis also show symptoms of obesity and insulin resistance, both conditions closely related to T2DM (38, 39). The prevalence of overweight and obesity in PCOS is significantly greater than that of the general female population. Impaired glucose tolerance in women with PCOS was found to be 3-fold higher than in women of similar age by the National Health and Nutrition Survey (NHANES) II. When age and weight were controlled, the comparative prevalence was two-fold (39). Even lean women with PCOS show an increased risk for T2DM (40). The risk for women suffering from both PCOS and psychosis that requires antipsychotic treatment is, therefore, very high.

Antipsychotic drugs predispose to metabolic syndrome (37), to which women with PCOS are already susceptible (41). The available evidence shows that dyslipidemia is very common in PCOS (42), and that elevated values of triglycerides and total and low-density lipoprotein-cholesterol are frequently present. It is well known that dyslipidemia, obesity, and diabetes are all potent cardiovascular risk factors, but it is not currently certain whether the increased cardiovascular risk seen in PCOS is mediated through obesity or through other metabolic factors. Despite uncertainty about the pathway, the risk of cardiovascular illness is significantly elevated in PCOS (43), as it is in patients with psychosis.

Cardiovascular disease is the major cause of mortality in patients with psychosis. The rate is approximately two times higher than it is in the general population (44) and antipsychotic drugs are, at least in part, responsible (45, 46). The similarities between both metabolic and cardiovascular side effects of patients in treatment for psychosis and patients with PCOS have profound health implications for women who suffer from both conditions.

Valproate can be used together or alone to treat bipolar disorder and it is known to induce PCOS-like symptoms. Therefore, patients maintained chronically on valproic acid should be monitored to avoid the development of menstrual irregularities and signs of PCOS, since the reproductive endocrine effects of valproate are reversible after the treatment is discontinued (47).

## Conclusions and Recommendations

Although PCOS is the most frequent of all endocrine disorders among women of reproductive age, many women do not receive adequate treatment because of a too late diagnosis. To facilitate accurate diagnosis and timely treatment, clinicians who see female patients need to be familiar with the diversity of PCOS phenotypes. Patients with severe mental illness, on the other hand, have limited access to physical healthcare services (48). For this reason, it is important that psychiatrists be aware of the possibility of PCOS in their patients. When they learn about menstrual cycle irregularity in their women patients or find signs of hyperandrogenism, such as acne, hirsutism, and acanthosis nigricans (49)*, they must not automatically attribute them to antipsychotic drug effects*. Routine referral for a gynecology/endocrinology consult is indicated.

Moreover, for women with PCOS and psychosis, treatment with antipsychotic drugs can worsen PCOS symptomatology and lead to negative consequences for a woman's reproductive potential and her quality of life. Antipsychotic-induced weight gain is an important concern in the management of these patients. Prevention of weight gain (choosing the right drug, keeping the dose as low as possible, instruction about diet, exercise, and substance use) is more effective than after-the-fact attempts at intervention. Adequate monitoring of body mass index, fasting glucose, and prolactin levels in patients on antipsychotics is vital for patients suffering from both conditions. Given the seriousness of psychotic conditions in women with PCOS, further study of epidemiology, clinical features, neurobiology, disability, quality of life, and treatment in different settings is needed to more fully understand this association.

## Author Contributions

LD, FM, and AC reviewed literature and wrote this paper.

## Conflict of Interest

The authors declare that the research was conducted in the absence of any commercial or financial relationships that could be construed as a potential conflict of interest.
